# *GmFT2a*, a Soybean Homolog of *FLOWERING LOCUS T*, Is Involved in Flowering Transition and Maintenance

**DOI:** 10.1371/journal.pone.0029238

**Published:** 2011-12-14

**Authors:** Hongbo Sun, Zhen Jia, Dong Cao, Bingjun Jiang, Cunxiang Wu, Wensheng Hou, Yike Liu, Zhihong Fei, Dazhong Zhao, Tianfu Han

**Affiliations:** 1 The National Key Facility for Crop Gene Resources and Genetic Improvement and MOA Key Lab of Soybean Biology (Beijing), Institute of Crop Science, The Chinese Academy of Agricultural Sciences, Beijing, China; 2 College of Life and Environmental Science, Minzu University of China, Beijing, China; 3 Department of Biological Sciences, University of Wisconsin Milwaukee, Milwaukee, Wisconsin, United States of America; Korea University, Republic of Korea

## Abstract

**Background:**

Flowering reversion can be induced in soybean (*Glycine max* L. Merr.), a typical short-day (SD) dicot, by switching from SD to long-day (LD) photoperiods. This process may involve florigen, putatively encoded by *FLOWERING LOCUS T* (*FT*) in *Arabidopsis thaliana*. However, little is known about the potential function of soybean *FT* homologs in flowering reversion.

**Methods:**

A photoperiod-responsive *FT* homologue *GmFT* (renamed as *GmFT2a* hereafter) was cloned from the photoperiod-sensitive cultivar Zigongdongdou. *GmFT2a* gene expression under different photoperiods was analyzed by real-time quantitative PCR. *In situ* hybridization showed direct evidence for its expression during flowering-related processes. *GmFT2a* was shown to promote flowering using transgenic studies in *Arabidopsis* and soybean. The effects of photoperiod and temperature on *GmFT2a* expression were also analyzed in two cultivars with different photoperiod-sensitivities.

**Results:**

*GmFT2a* expression is regulated by photoperiod. Analyses of *GmFT2a* transcripts revealed a strong correlation between *GmFT2a* expression and flowering maintenance. *GmFT2a* transcripts were observed continuously within the vascular tissue up to the shoot apex during flowering. By contrast, transcripts decreased to undetectable levels during flowering reversion. In grafting experiments, the early-flowering, photoperiod-insensitive stock Heihe27 promotes the appearance of *GmFT2a* transcripts in the shoot apex of scion Zigongdongdou under noninductive LD conditions. The photothermal effects of *GmFT2a* expression diversity in cultivars with different photoperiod-sensitivities and a hypothesis is proposed.

**Conclusion:**

*GmFT2a* expression is associated with flowering induction and maintenance. Therefore, *GmFT2a* is a potential target gene for soybean breeding, with the aim of increasing geographic adaptation of this crop.

## Introduction

The timely transition to flowering is crucial for successful plant reproduction. As such, this process is controlled by both endogenous and environmental signals. *Arabidopsis thaliana* contains at least four flowering pathways that are responsive to these cues including the gibberellin, autonomous, photoperiod and vernalization flowering pathways [1]–[4]. Therefore, a complex flowering regulatory network exists that includes many known genes such as *FLOWERING LOCUS T* (*FT*), *CONSTANS (CO)*, *FLOWERING LOCUS C* (*FLC*), *APETALA1* (*AP1*), and *SUPPRESSOR OF OVEREXPRESSION OF CO 1* (*SOC1*) [5]–[7]. *FT*, the putative florigen gene, is central to this network. *FT* functions by integrating signals from various pathways to regulate flowering time [8], [9]. *FT* and its homologues, belonging to the FT-like subfamily of the PEBP (phosphatidylethanolamine binding domain) family, promote flowering [10], [11]. Members of the FT-like subfamily are most highly expressed in leaves in response to inductive photoperiods. In *Arabidopsis,* an inductive LD photoperiod activates *FT* expression to regulate flowering [12], [13] and there is a similar case for *Hd3a* in rice in inductive SD photoperiods [14]. From evidence of grafting experiments, FT protein can be transported from the leaves to the shoot apex to induce flowering [15]–[18]. It is therefore believed that either FT protein is itself florigen, or else it is a potential component of florigen [11], [18]–[20]. Li et al. [21] found that *FT* mRNA can mediate long-distance trafficking of heterologous RNAs, indicating that *FT* mRNA may also function in florigen transportation. However, the role it plays in doing so is still a conundrum. Therefore, *FT* plays an important role in flowering transition, but more details are needed to elucidate the mechanism of florigen transportation.

Flowering transition can be reversed. In some species, flowering reversion occurs after specific treatments [22]–[25]. Soybean is one such species, in which flowering will be reversed in response to LD. It is expected that *FT* should be involved in flowering reversion, and Molinero-Rosales et al. [26] demonstrated that the tomato *FT* mutant *sft* displays a flowering-reversion phenotype with some sepals converted into leaves in the first floral whorl. Nevertheless, details are quite absent and additional studies will be necessary to elucidate if and how *FT* functions in flowering reversion.

In addition to photoperiod, temperature is also an important environmental cue for successful reproduction. *FT* is also likely involved in this response. High temperatures promote *Arabidopsis* flowering via the autonomous pathway [27] and this effect is related to *FT* expression [28]–[31]. In *Arabidopsis*, *SHORT VEGETATIVE PHASE* (*SVP*) mediates the response to ambient temperatures through the negative regulation of *FT* expression [31], [32]. In *Satsuma mandarin*, low temperature specifically affected *CiFT* transcripts in adult plants but not in juveniles [33]. The *Arabidopsis phyB* mutant exhibits a temperature-sensitive precocious-flowering phenotype [29]. It is suggested that flowering is controlled by interactions between photoperiod and temperature, although the underlying mechanism remains unclear.

Soybean, an SD dicot crop of economic and agricultural importance, includes many cultivars with diverse photoperiod-sensitivities. The photoperiod-sensitive late-flowering cultivar Zigongdongdou (from Zigong, Sichuan Province in south China) does not flower during long days or in northern regions [34]. The photoperiod-insensitive early-flowering cultivar Heihe27 (from Heihe, Heilongjiang Province in northeast China) fails to produce high yields in southern regions due to the high temperatures that occur there [35]. Following a shift from SD to LD, flowering reversion will occur in Zigongdongdou, but not in Heihe27 [24], [25], [36]. With Zigongdongdou as a model plant, Wu et al. [25] established an effective experimental system to study flowering mechanisms in both forward and reverse directions. Therefore, these two cultivars are very suitable models for addressing questions about the mechanisms involved in flowering transition and maintenance.

To date, based upon the soybean genome draft sequence [37], two reports have been made concerning *FT*-like genes in the soybean genome [10], [38]. Thakare et al. [38] found nine *FT*-like genes: *Glyma16g26660*, *Glyma16g26690*, *Glyma08g47810*, *Glyma08g47820*, *Glyma18g53690*, *Glyma18g53680*, *Glyma16g04830*, *Glyma19g28400* and *Glyma19g28390*. Two of these genes, *Glyma16g26660* and *Glyma16g04830*, can be detected by real-time quantitative PCR and their expression is inhibited by LD photoperiods. Kong et al. [10] identified an additional *FT*-like gene (*Glyma16g04840*), cloned six of the ten *FT*-like genes, and confirmed that transgenic overexpression of *GmFT2a* (*Glyma16g26660*) and *GmFT5a* (*Glyma16g04830*) can promote precocious flowering in *Arabidopsis*. *GmFT2a* and *GmFT5a* respond differently to photoperiod, and PHYA may be involved [10]. However, the relationship between these two genes and the flowering process need to be further elucidated.

This study reports the cloning of a new soybean *FT* homologue *GmFT* (later renamed to *GmFT2a* as Kong et al. first designated [10]) and the characterization of its expression using real-time quantitative PCR and *in situ* hybridization during flowering and flowering reversion. The ability of *GmFT2a* to promote flowering in *Arabidopsis* and soybean was analyzed. Additionally, *GmFT2a* expression in Zigongdongdou and Heihe27 grown under different photoperiods and temperatures was examined. The results indicate that *GmFT2a* may play an important role, not only in flowering transition, but also in flowering maintenance. Furthermore, the impact of temperature on *GmFT2a* is dependent on photoperiod sensitivity.

## Results

### Molecular Cloning and bioinformatic analysis of *GmFT*

*GmFT* (Genbank accession number EU287455), a soybean homolog of *FT*, was cloned, based on an EST isolated from a Zigongdongdou SSH library. *GmFT* contains 888 base pairs (bp) and encodes a protein of 176 amino acids (shown in Supporting Information S1). GmFT includes a conserved PEBP domain and belongs to the FT-like subfamily of the PEBP family. To date, the soybean genome data is available at http://www.phytozome.net/soybean.php. This genome contains at minimum nine, and possibly ten, putative soybean *FT*-like genes [10], [38]. By aligning *GmFT* with *Glyma16g26660* (shown in Supporting Information S1), *GmFT* was shown to fully contain the *Glyma16g26660* putative transcript, i.e., *GmFT2a* as described by Kong et al. [10], therefore, *GmFT* was renamed to *GmFT2a* hereafter although *GmFT* contains 5′ and 3′ UTR sequences which are absent from *GmFT2a* [10]. Shown in Supporting Information S1, *GmFT2a* has a conserved gene structure with other *FT*s. GmFT2a has greater sequence similarity to FT than to TFL1, especially in the external loop related to the FT/TFL activity [39]. A key amino acid residue, Tyr85 (FT)/His88 (TFL1), differentiates the activities of FT and TFL1 [40]. GmFT2a contains the same Tyr85 as FT. Therefore, it is reasonable to hypothesize that *GmFT2a* can promote flowering as *FT* does.

### *GmFT2a* is primarily expressed in leaves and regulated by photoperiods

**Figure 1:**
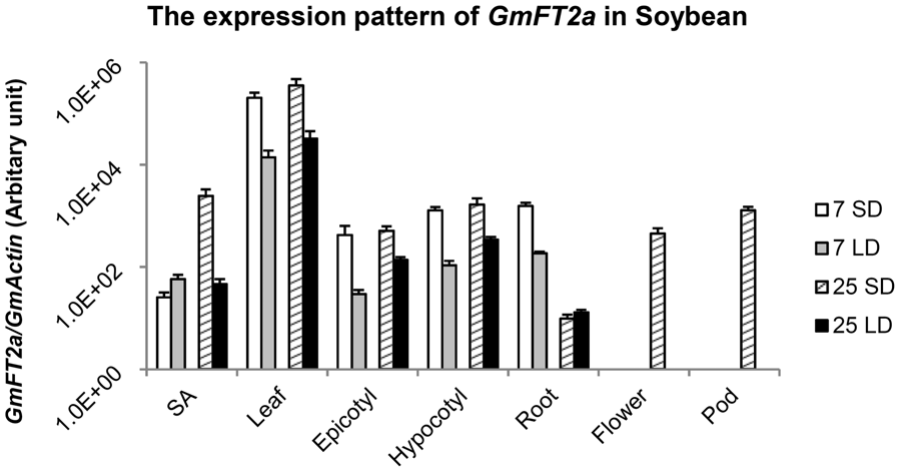
The expression pattern of *GmFT2a* in soybean. The expression level of *GmFT2a* in different tissues or organs was detected by real-time quantitative PCR at 7 or 25 days after the first pair of unifoliate leaves fully expanded under short days (SD) or long days (LD). *GmACTIN* was used as a control gene. The data of flower and pod were not available for 7 SD, 7 LD or 25 LD, as cultivar Zigongdongdou did not flower at that time. SA, shoot apex (not including any manually removable leaves). For each real-time quantitative PCR point, the averages and standard errors are the result of three replications.

Seeds of the cultivar Zigongdongdou were planted and maintained under LD conditions until the unifoliates were fully expanded. One set of plants were then grown under LD conditions and another set under SD conditions. In the course of the experiment, the plants grown under SD flowered at the 25th day of this condition, but the plants under LD did not flower. *GmFT2a* expression was profiled with real-time quantitative PCR using *GmActin* as a reference. *GmFT2a* expression in the leaves was higher than in other tissues or organs. Moreover, inductive SD photoperiods significantly increased *GmFT2a* expression with the exception of 7-day-treated shoot apices or 25-day-treated roots (Figure 1). These results are consistent with *FT* [41], *GmFT2a* and *GmFT5a* [10]. Therefore, photoperiod may regulate *GmFT2a* expression to control the soybean flowering process. Notably, *GmFT2a* was detected in both flowers and pods (Figure 1), suggesting that *GmFT2a* may function in reproductive development.

### LD treatments postpone the diurnal peak of *GmFT2a* gene expression

**Figure 2:**
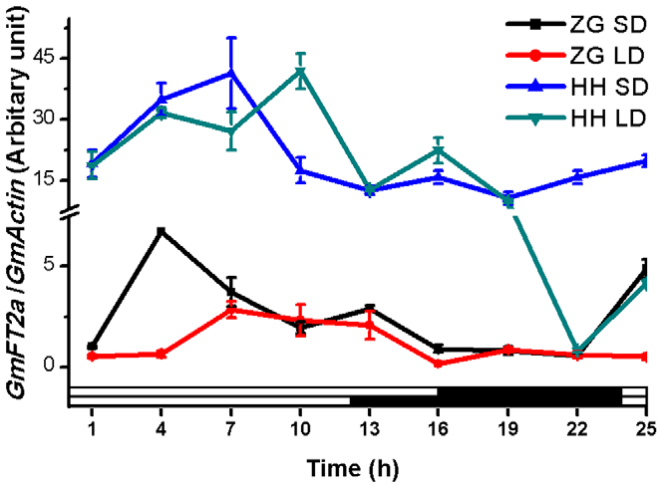
The diurnal circadian rhythm of *GmFT2a* gene expression under SD and LD conditions. Unifoliate leaves were sampled every 3 hours since 1 hour after dawn. White bars at the bottom represent light phases, and black bars dark phases. ZG SD, Zigongdongdou under SD conditions; ZG LD, Zigongdongdou under LD conditions; HH SD, Heihe27 under SD conditions; and HH LD, Heihe27 under LD conditions.

From two cultivars, late-flowering photoperiod-sensitive Zigongdongdou and early-flowering photoperiod-insensitive Heihe27, unifoliate leaves were sampled every 3 hours since 1 hour after dawn. We isolated RNA from these leaves and analyzed the diurnal circadian rhythm of *GmFT2a* gene expression by quantitative real-time PCR (Figure 2). In Zigongdongdou, SD induced the expression of *GmFT2a*, consistently with previous results. In Heihe27, the gene expression level was higher than in Zigongdongdou under both photoperiods, which could explain the fact that Heihe27 is early-flowering and photoperiod-insensitive. For these two cultivars, *GmFT2a* exhibited a diurnal circadian rhythm both under SD and LD conditions. Under SD conditions, *GmFT2a* gene expression reached a peak 4 h and 7 h after dawn for Zigongdongdou and Heihe27 respectively, which was similar with the results of Kong et al. [10]. Under LD conditions, the expression peak was postponed for about 3 hours for both cultivars. It was suggested that *GmFT2a* gene expression might be regulated by circadian clock.

### *GmFT2a* accompanies flower development

**Figure 3:**
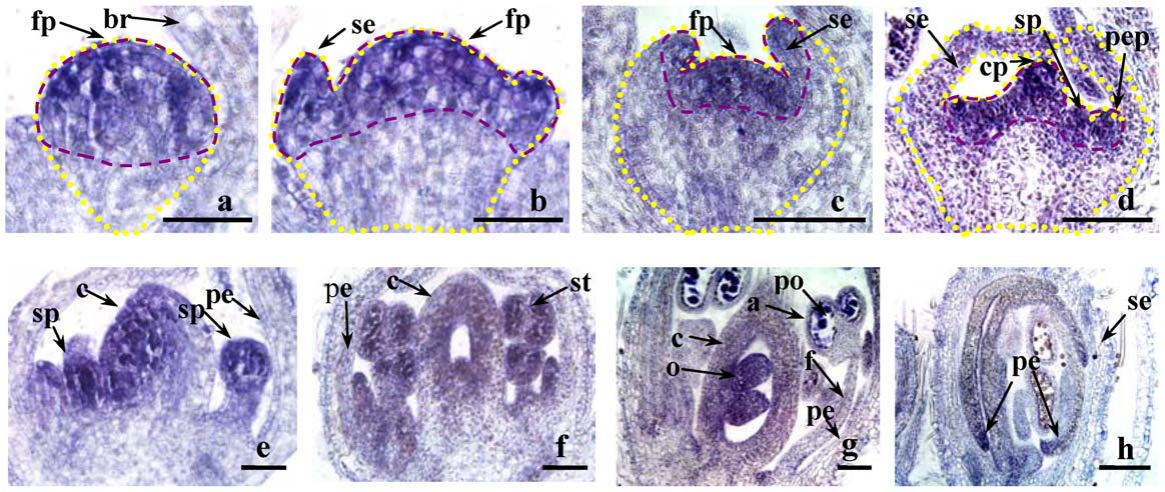
*GmFT2a* expression during flower development. (**a**) Global floral primordia. (**b**) Floral primordia with emerging sepals. (**c**) Floral primordia with sepals. (**d**) Flower structure initially formed with primordia of petal, stamen and pistil. (**e**) Developing carpel and stamen. (**f**) Initially formed ovary and stamen. (**g**) Ovary with ovules and developed stamen with pollen. (**h**) Flower bud. The profile of the flower primordia is enclosed with yellow dashes, and the GmFT2a signals are contained mainly in the region enclosed with red dashes. a, anther; br, bract; c, carpel; cp, carpel primordium; f, filament; fp, flower primordium; p, pistil; pe, petal; po, pollen; se, sepal; sp, stamen primordium; st, stamen. Bar, 250 µM.

*GmFT2a* expression during flowering was analyzed using *in situ* hybridization. *GmFT2a* transcripts initially appeared in the upper portion of the global floral primordia (Figure 3a) and then spread to the sepals and sepal primordia (Figure 3b). During sepal development, *GmFT2a* expression gradually decreased from the base to the apex of young sepals (Figure 3c). When the primordia of petals, stamens and carpels appeared, the *GmFT2a* levels of these primordia were robust (Figure 3d) while the *GmFT2a* levels in the young petals were attenuated (Figure 3e). *GmFT2a* transcripts at the stamen apex were detected throughout stamen development. During the development of the carpel, the *GmFT2a* transcripts gradually accumulated in the inner carpel but attenuated at the carpel periphery (Figure 3e,f). *GmFT2a* expression at the ovary wall disappeared gradually but increased in the new-borne ovules (Figure 3f,g). *GmFT2a* transcripts were also present at the edge of the petal (Figure 3h). These results indicate that during flower development, *GmFT2a* transcripts are continuously present in the primordia of flower organs where cell division is vigorous. These data also suggest that *GmFT2a* plays important roles in flower development.

### LD induces flowering reversion with *GmFT2a* expression decreasing

**Figure 4:**
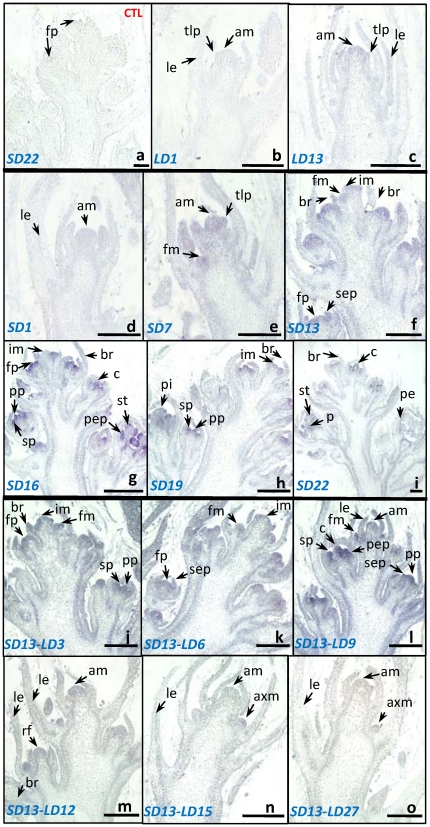
The expression pattern of *GmFT2a* in soybean cultivar Zigongdongdou shoot apices under different photoperiod conditions by *in situ* hybridization. (a) Shoot apex detected by the sense *GmFT2a* probe as a control. All other shoot apices were detected by the antisense *GmFT2a* probe shown in (b)–(o). (a) Shoot apex at the 22nd day of SD conditions (briefed as SD22). (b) and (c) Shoot apexes at LD1 and LD13, respectively. (d)–(i) Shoot apices at SD1, SD7, SD13, SD16, SD19, and SD22, respectively. (j)–(o) Shoot apices treated by LD for 3, 6, 9, 12, 15, and 27 days after SD13, respectively. am, apical meristem; axm, axillary meristem; br, bract; c, carpel; fm, floral meristem; fp, floral primordium; im, inflorescence meristem; ip, inflorescence primordium; le, leaf/trifoliolate leaf; p, pistil; pe, petal; pep, petal primordium; pp, pistil primordium; rf, reversed flower; s, stamen; se, sepal; sep, sepal primordium; sp, stamen primordium; tlp, trifoliolate leaf primordium. Bar, 250 µM.

*GmFT2a* expression was analyzed by *in situ* hybridization in Zigongdongdou under different photoperiods. Under LD conditions, Zigongdongdou retains its vegetative growth [36]. Consistent with this, *GmFT2a* transcripts were barely detected in the apical or lateral meristems at the 1st or 13th LD (briefed as LD1 and LD13, respectively, Figure 4b,c). Under SD conditions, Zigongdongdou flowers approximately 29 days after cotyledon expansion [36]. Although *GmFT2a* transcripts were not detected at the 1st SD (abbreviated SD1, Figure 4d), they did appear slightly in the apical and lateral meristems at SD7. The lateral floral meristems also appeared at SD7, indicating that the flowering transition had already occurred (Figure 4e). At SD13, inflorescence differentiation was initiated at the shoot apices as indicated by the formation of small and slim bracts, the appearance of sepal primordia, and the enlargement of the floral meristems and primordia. Consistent with the timing of inflorescence differentiation, *GmFT2a* transcripts appeared in the primordia of the flowers, sepals and pistils and strengthened in the floral meristems (Figure 4f). At SD16 and SD19, the terminal inflorescences were still differentiating with the floral meristems, petals, pistils and stamens being produced. During this time, *GmFT2a* was strongly detected in these developing floral meristems, organs and primordial, but weakly in the inflorescence meristems (Figure 4g,h). At SD22, *GmFT2a* appeared in the floral primordia and organs but attenuated, even disappeared, in the stamens and petals of developed flowers at their inflorescence base (Figure 4i). It is notable that the *GmFT2a* transcripts were consistently detected in the vascular tissues (Figure 4d–i), suggesting that *GmFT2a* transcripts might be consecutively transcribed locally or transported through vascular system to meristems and primordia. Zigongdongdou flowering is reversed if plants are shifted to LD conditions after 13 days of SD [25]. As shown in Figure 4j–l, *GmFT2a* expression was intense in the floral meristems and floral organ primordia at the 3rd, 6th, and 9th LDs after SD13 (abbreviated SD13–LD3, SD13–LD6, and SD13–LD9, respectively), with terminal inflorescence differentiation still occurring until SD13–LD9. At SD13–LD9, the inflorescence meristems were reversed to vegetative meristems with small stipules and trifoliolates formed (Figure 4l). At SD13–LD12, the shoot apices were completely shifted into vegetative growth and one reversed flower was formed. However, no *GmFT2a* transcripts were detected (Figure 4m), suggesting that LD-induced flowering reversion is related to *GmFT2a* attenuation. At SD13–LD15 and SD13–LD27, *GmFT2a* levels were significantly decreased, even undetectable, in stem apices (Figure 4n,o). These results indicate that *GmFT2a* transcripts are correlated with the maintenance of flower differentiation.

### Early-flowering stock Heihe27 promotes the appearance of *GmFT2a* transcripts in the late-flowering scion Zigongdongdou

**Figure 5:**
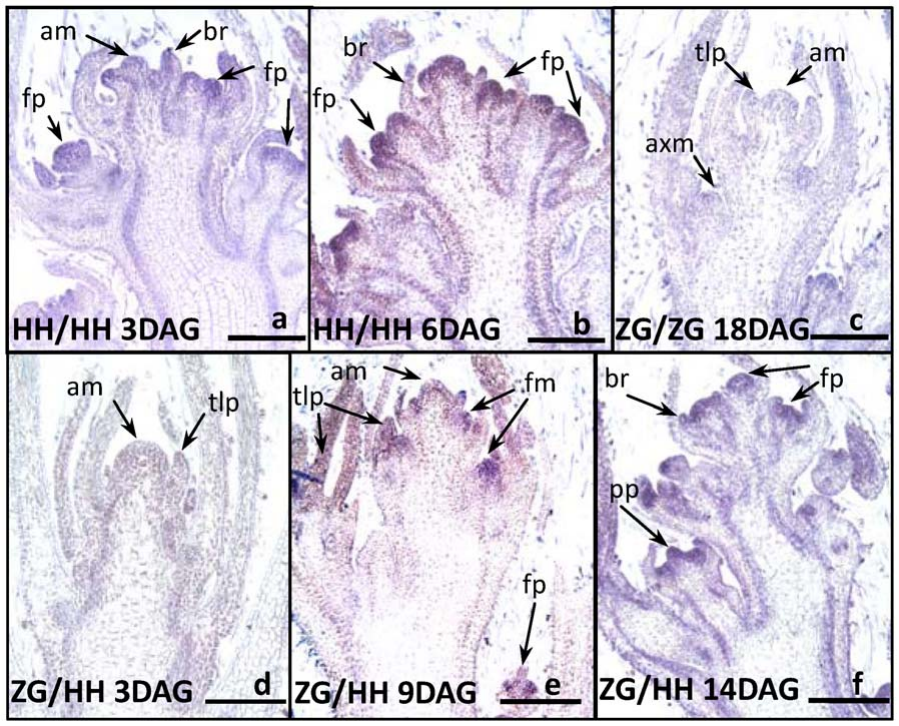
Early-flowering stock promotes the appearance of *GmFT2a* transcripts in late-flowering scion by grafting. (**a–b**) Shoot apices of self-grafted Heihe27 (HH/HH, scion/stock) at the 3rd or 6th day after grafting (DAG), respectively. (**c**) Shoot apex of self-grafted Zigongdongdou (ZG/ZG) at 18 DAG. (**d–f**) Shoot apices of scion Zigongdongdou grafted on stock Heihe27 (ZG/HH) at 3, 9 or 14 DAG, respectively. am, apical meristem; axm, axillary meristem; br, bract; fm, floral meristem; fp, floral primordium; pp, pistil primordium; tlp, trifoliolate leaf primordium. Bar, 250 µM.

Flowering occurs in self-grafted Heihe27 (HH/HH, scion/stock) under LD conditions. Floral differentiation began 3 days after grafting (3 DAG). Accordingly, *GmFT2a* transcripts were initially detected in flower primordia at 3 DAG with transcript abundance increasing at 6 DAG in concurrence with floral differentiation (Figure 5a,b). Under the experimental LD conditions of this study, self-grafted Zigongdongdou (ZG/ZG) did not flower. Consistently, ZG/ZG scions continued their vegetative growth with trifoliates produced continuously up to 18 DAG. In these plants, *GmFT2a* transcripts were rare in both apical and axillary meristems (Figure 5c). However, when the scion Zigongdongdou was grafted onto the stock Heihe27 (ZG/HH), flower differentiation occurred in the scion under LD. At 3 DAG, ZG/HH did not initiate floral meristems and *GmFT2a* transcripts were hardly detected in the scion apical meristems (Figure 5d). By 9 DAG, flower differentiation had been initiated and *GmFT2a* transcripts were apparent in flower meristems and primordia (Figure 5e). The amount of *GmFT2a* transcripts continued to increase up to 14 DAG (Figure 5f). These results suggest that the early-flowering stock Heihe27 is able to promote *GmFT2a* appearance in the late-flowering scion Zigongdongdou, prompting the scion to flower.

### Transgenically overexpressed *GmFT2a* promotes flowering

**Figure 6:**
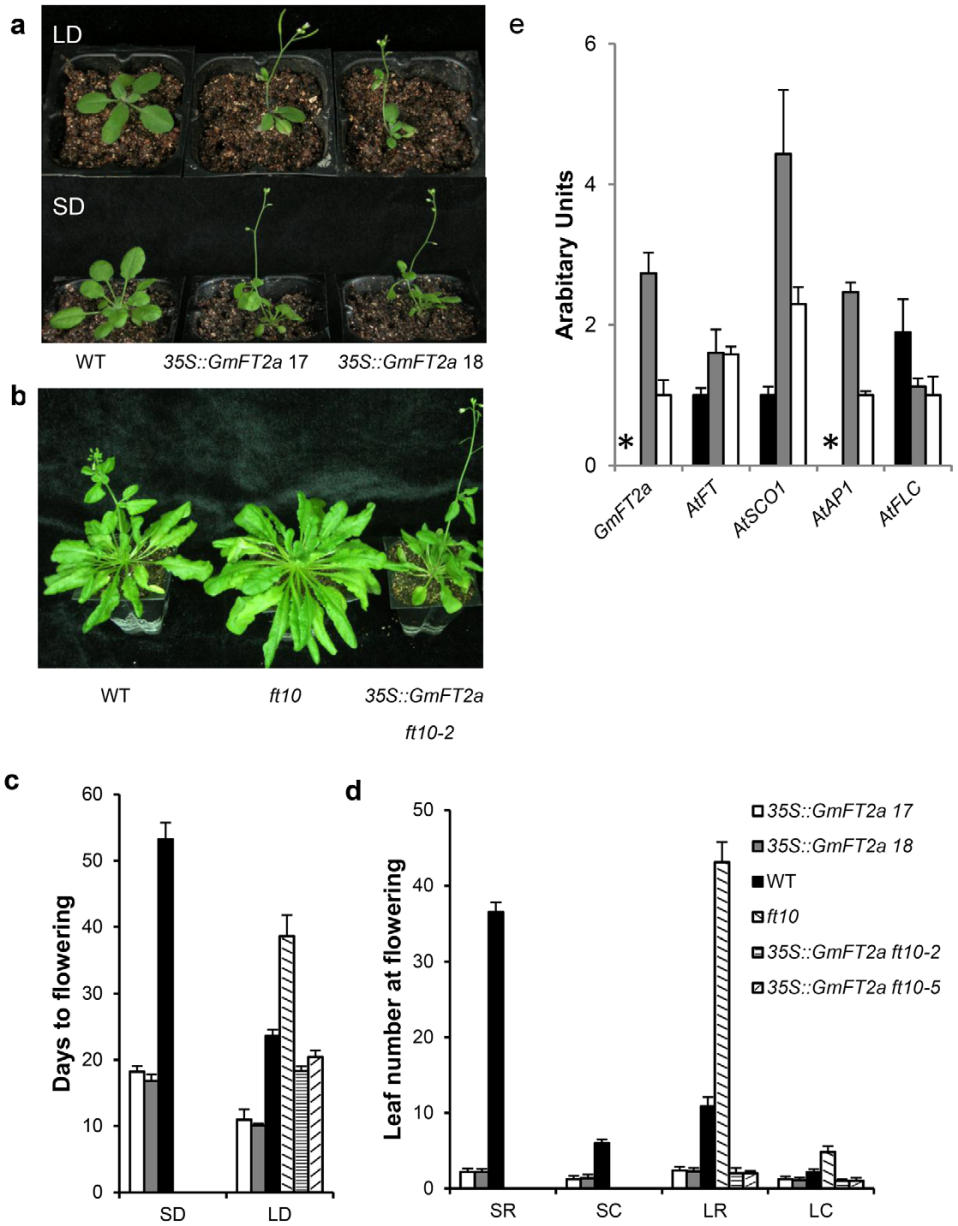
Transgenic analysis of *GmFT2a* in *Arabidopsis thaliana*. (**a**) Upper, *Arabidopsis* plants grown under long days (LD); Lower, *Arabidopsis* plants grown under short days (SD) when the transgenic plants flowered. From left to right, plants represent wildtype, transgenic line 17 and 18. (**b**) Transgenic overexpression of *GmFT2a* fully suppresses the late-flowering phenotype of a *FT* mutant *ft10*. From left to right, plants represent wild type, mutant *ft10*, and transgenic line 2 with mutant *ft10* genetic background. (**c**) The days to flowering of *Arabidopsis* plants. (**d**) The number of cauline and rosette leaves at flowering. SR, rosette leaves under SD conditions. SC, cauline leaves under SD conditions. LR, rosette leaves under LD conditions. LC, cauline leaves under LD conditions. (**e**) Detection of *GmFT*, *AtFT*, *AtAP1*, *AtSOC1*, and *AtFLC* by real-time quantitative PCR. Asterisks show that the data was not available at flowering. For each real-time quantitative PCR point, averages and standard errors are the result of three replications.

**Figure 7:**
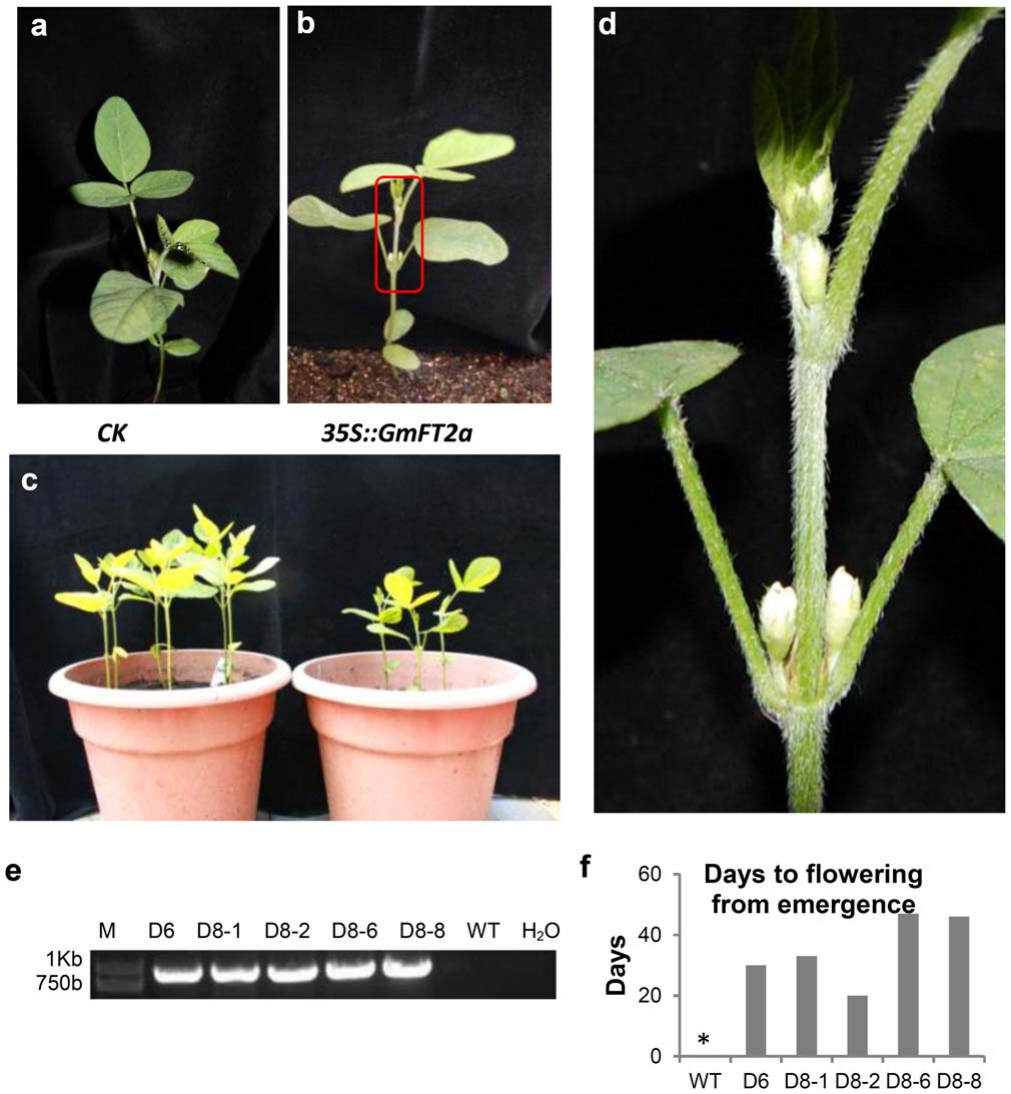
Transgenic *GmFT2a* induces precocious flowering in soybean cultivar Zigongdongdou. (**a**) A wild-type Zigongdongdou plant. (**b**) A transgenic Zigongdongdou plant showing precocious flowering at the axils of the unifoliate leaves. (**c**) The overview of wild-type (left) and transgenic (right) plants, including the plants shown in (**a**) and (**b**). (**d**) The close shot of the transgenic plant in (**b**) shows the precocious flowers at the axils of the unifoliate leaves. (**e**) Identification of transgenic *GmFT2a* plants by reverse transcription PCR with primers specific to *35S::GmFT*. (**f**) Days to flowering from emergence of the transgenic plants and wild-type plants. The asterisk indicates that wild-type plants cannot flower at LD conditions during this experiment.

To confirm whether *GmFT2a* promotes flowering, *GmFT2a* driven by the 35S cauliflower mosaic virus (CaMV) promoter was transformed into *Arabidopsis* and its *FT* mutant *ft10* [13]. Regardless of genetic backgrounds and photoperiods, plants overexpressing *GmFT2a* did promote flowering with few leaves (Figure 6a–d). Shown in Figure 6b,c, exogenously produced *GmFT2a* completely rescued, even over-compensated, *ft10* [13]. Therefore, *GmFT2a* function may be similar to *FT*. Real-time quantitative PCR of the transgenic lines revealed that some flowering-related genes (*FT*, *SOC1*, and *AP1*) were induced while *FLC* was suppressed. As expected, *GmFT2a* was expressed only in the transgenic lines (Figure 6e). Furthermore, overexpressed *GmFT2a* also promoted the flowering of Zigongdongdou even under noninductive LD conditions (Figure 7). One transgenic line flowered approximately 20 days after emergence. By contrast, the early-flowering Heihe27 flowers approximately 27 days after emergence. At the first flowering, the transgenic line contained few leaves, only one pair of unifoliates and one trifoliate. In this line, the flowers were located at the axils of the unifoliates, a location where flowers are not typically produced in this and most other soybean cultivars (Figure 7b,d). These results demonstrate that *GmFT2a* overexpression can promote precocious flowering independent of the photoperiod.

### Temperature impacts *GmFT2a* expression in a genotype-dependent manner

**Figure 8:**
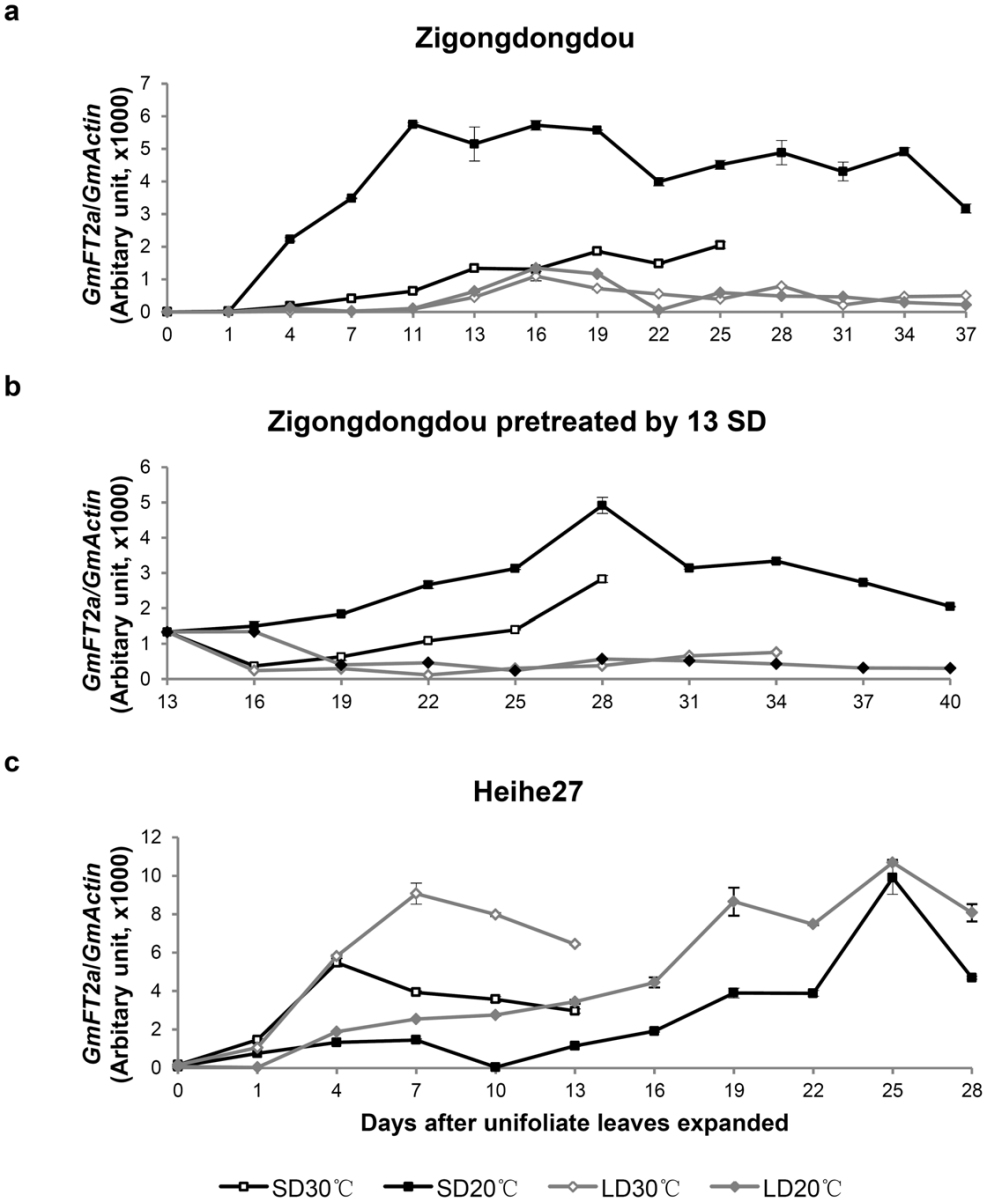
*GmFT2a* expression under different combinations of photoperiod and temperature. *GmFT2a* expression was detected in Zigongdongdou (**a**), Zigongdongdou pretreated by 13 SD (**b**), and Heihe27 (**c**) under short days at 30°C (SD30°C) or 20°C (SD20°C), and under long days 30°C (LD30°C) or 20°C (LD20°C). Zigongdongdou plants, including pretreated plants, did not flower throughout this experiment under LD conditions. For all other treatments, sampling ceased when the plants began blooming. For each real-time quantitative PCR point, averages and standard errors are the result of three replications.

Characterization of the *GmFT2a* expression of cultivars grown under varying photoperiods and temperatures will help elucidate *GmFT2a* functions and ultimately benefit soybean breeding. The photoperiod-sensitive Zigongdongdou flowered approximately 24 days after the unifoliates fully expanded (24 DAUE) under SD conditions and a temperature of 30°C (SD30°C). Flowering occurred at approximately 36 DAUE under SD20°C. However, Zigongdongdou did not flower under LD. Under SD conditions, *GmFT2a* transcripts increased gradually with higher expression noted under SD20°C compared to SD30°CC Under LD conditions, *GmFT2a* transcripts were rarely detected and no significant differences were observed between LD30°C and LD20°C (Figure 8a). Similar results were obtained when Zigongdongdou was pretreated by 13 SD (Figure 8b). These results indicate that under inductive photoperiods, low temperature promotes *GmFT2a* expression in Zigongdongdou. Under noninductive photoperiods, temperature does not regulate *GmFT2a* expression (Figure 8a,b). The photoperiod-insensitive Heihe27 flowered approximately 15 DAUE under SD30°C, 38 DAUE under SD20°C, 18 DAUE under LD30°C, and 40 DAUE under LD20°C. *GmFT2a* also increased gradually, peaked, and then decreased slowly (Figure 8c). Notably, *GmFT2a* expression was higher under LD when compared to SD grown at the same temperature. *GmFT2a* expression was greater at high temperature compared to low temperature when grown under the same photoperiod (Figure 8c). Therefore, photoperiod is hypothesized to play a dominant role in *GmFT2a* expression in photoperiod-sensitive cultivars while temperature is suspected to play an important role in *GmFT2a* expression in photoperiod-insensitive cultivars.

## Discussion

Soybean is not only a typical SD crop of high economic importance, but is also an excellent model to elucidate flowering mechanisms using both forward and reverse approaches. Using soybean as a model to study the role of *FT* in flowering reversion will lead to a greater understanding of the flowering mechanism.

### *GmFT2a* promotes flowering

The *FT* homologue described in this paper contains 5′ and 3′ UTRs that are not present in the *GmFT2a* Kong et al. reported before [10]. An analysis of the role of the UTRs in the function of *GmFT2a* may provide interesting results as a PlantCARE search revealed that the 3′UTR contains a motif associated with the circadian mRNA accumulation [42]. Shown in Figure 2, for both Zigongdongdou and Heihe27, *GmFT2a* expression exhibits a diurnal circadian rhythm and LD can postpone the expression peak, suggesting that photoperiod might regulate the circadian peak time of *GmFT2a* expression to control flowering time. Consistent with previous studies, transgenic overexpression of *GmFT2a* not only promotes flowering of *Arabidopsis* and the soybean cultivar Zigongdongdou but also over-compensates the *ft10* phenotype (Figure 6,7) [8]–[10], [43]. Thus, *GmFT2a* may encode florigen or a component of florigen in soybean. However, there are numerous other *FT*-like genes in the soybean genome, and at least one of them (*Glyma16g04830*, *GmFT5a*) has also been confirmed to promote flowering [10]. Therefore, why soybean contains so many FT-like genes and how they function and interact must be further studied before the soybean flowering mechanisms can be elucidated.

### *GmFT2a* is related to flowering maintenance

In addition to promoting flowering transition, *GmFT2a* may play a role in subsequent flower development. *In situ* hybridization identified the continuous presence of *GmFT2a* transcripts in all primordia during flower development (Figure 3,4). Further indications arose from the results of flower reversion induced by LD treatment. As shown in Figure 4, LD treatment could neither terminate flower differentiation nor decrease *GmFT2a* transcripts immediately, possibly a consequence of the preceding SD treatment [34]. As LD treatment continued, *GmFT2a* transcripts attenuated gradually. Additionally, flower differentiation first slowed down and then terminated before leaf differentiation launched, meaning flowering reversion had occurred (Figure 4). Consistent with Molinero-Rosales et al. [26], these results suggest that *GmFT2a* is involved in flowering maintenance. On one hand, the maintenance of the identity of floral meristems may be dependent on the durative expression of *GmFT*. On the other hand, flowering reversion induced by LD treatment may decrease or degrade *GmFT2a* mRNA. Given that transgenically overexpressed *GmFT2a* promoted soybean flowering under LD conditions, it is more reasonable that *GmFT2a* maintains floral identity, although further investigation is needed. Furthermore, whole plant reversion occurs when soybean plants are transferred to LD conditions even after flowering under SD, indicating that soybean responds to photoperiods both before and after flowering [23], [24], [44], [45]. Further study of the role of *GmFT2a* in whole plant reversion will lead to a better understanding of florigen.

### *GmFT2a* transcripts consecutively appear along vascular tissues to the primordia

As reviewed by Turck et al. [6], a discrepancy exists. That is, *FT* RNA is only expressed in vascular tissues [46], [47] and FT protein functions in meristems [41], [48]. With evidence from grafting studies, FT protein can be transported from the leaves to the shoot apex to induce flowering [15]–[18]. It is believed that the FT protein could be florigen or a component of florigen [11], [18]–[20]. To date, there is little direct evidence concerning the spatial distribution of *FT* expression using techniques like *in situ* hybridization. Because 10 *FT*-like genes exist in the soybean genome and six of these genes are transcribed [10], *GmFT*-specific probes are necessary. Based on sequence alignments, there are no significant similarities between the *GmFT2a* 3′ UTR and other genes (including the other nine *FT*-like genes) in the soybean genome (data not shown); therefore, probes recognizing the *GmFT2a* 3′ UTR are gene-specific. *In situ* hybridization using *GmFT*-specific probes revealed the presence of *GmFT2a* transcripts in vascular tissues as well as meristems and primordia (Figure 4). Therefore, *GmFT2a* is different to *FT*, *Hd3a* and *SFT* [46], [47], [49], [50]. There are at least two possibilities: *GmFT2a* could be consecutively transcribed locally, or *GmFT2a* could be transported along the vascular tissues to the primordia. These possibilities may exist given that soybean is an SD dicot, and thus, different from *Arabidopsis* (an LD dicot), rice (an SD monocot), and tomato (a day-neutral dicot). There may be differences in the mechanism of florigen transportation and activity between species. Recently, Mimida et al. found that in apple (*Malus* x *domestica* Borkh.), *MdFT*, a *FT* homolog, is expressed in the fruit-bearing shoot apex during the flower induction period and suggested that MdFT protein might not necessarily function as a transmissible floral inducer [51]. Their results seem to support our hypothesis. In some species, RNA may be the major, or most efficient form for florigen transportation; in other species, protein may be the major or most efficient form. In any case, more research is necessary before these florigen-related questions are answered.

By grafting, the stock Heihe27 promoted the appearance of *GmFT2a* in the shoot apices of the scion Zigongdongdou under LD conditions (Figure 5). However, *GmFT2a* expression was significantly inhibited by LD and nearly undetectable in the shoot apices of ungrafted and self-grafted Zigongdongdou grown under the same conditions. Given the rather high level of *GmFT2a* expression in Heihe27 under LD conditions (Figure 8), it is reasonable to hypothesize that the *GmFT2a m*RNA detected in the scion Zigongdongdou may be from stock Heihe27. As *FT* expression was induced in *GmFT*-overexpressing transgenic *Arabidopsis* (Figure 6), it is also possible that *GmFT2a* protein from the stock Heihe27 may activate the transcription of *GmFT2a* in the scion Zigongdongdou.

### *GmFT2a* expression is dependent upon photoperiod

*GmFT2a* expression is much higher in leaves than in other organs, consistent with the expression of *FT*, and *Hd3a* [9], [10], [41], [50]. The expression of *FT*s is promoted by inductive photoperiods and inhibited by noninductive photoperiods in *Arabidopsis* [9], rice [43] and soybean [10], [38]. Accordingly, in the photoperiod-sensitive cultivar Zigongdongdou, *GmFT2a* was induced by SD and suppressed by LD (Figure 1). However, *GmFT2a* expression was different in the photoperiod-insensitive Heihe27. Under SD, *GmFT2a* expression peaked earlier in Heihe27 compared to Zigongdongdou (Figure 8), coinciding with earlier flowering in Heihe27 compared to Zigongdongdou. These results suggest that the time of flowering may be related to the peak of *GmFT2a* expression. Under LD conditions, Heihe27 flowered while Zigongdongdou did not. This result was consistent with *GmFT2a* expression, which was suppressed in Zigongdongdou but not in Heihe27. Notably, in Heihe27, *GmFT2a* expression was higher under LD than SD conditions. Considering that Heihe27 flowered under LD conditions at the same time as under SD conditions, it is hypothesized that more *GmFT2a* is helpful for Heihe27 to overcome a potential flowering barrier imposed by the intrinsic noninductive photoperiod LD. Therefore, *GmFT2a* may be involved in the diversity of photoperiod-sensitivity in soybean cultivars.

### Photoperiod and temperature interactively regulate *GmFT2a* expression

Previous results indicated that photoperiod and temperature interact to control soybean development [52]; hence, it was hypothesized that they would also interact in the regulation of *GmFT2a* expression. In the photoperiod-sensitive cultivar Zigongdongdou grown under LD conditions (Figure 8), *GmFT2a* expression was suppressed significantly with temperature apparently playing no role. This result is consistent with the inability of Zigongdongdou to flower under LD but not SD conditions. High temperatures suppressed *GmFT2a* expression but promoted flowering of Zigongdongdou under SD. There are two potential reasons: high temperature may inhibit some flowering inhibitor(s), or high temperature may increase the transit efficiency of *GmFT2a* mRNA or protein from the leaves to the apical meristem. Other possibilities, however, cannot be discounted. For the photoperiod-insensitive Heihe27 grown under two temperature regimes (Figure 8), *GmFT2a* expression was always higher under LD in comparison to SD conditions possibly to overcome potential flowering barriers imposed by the noninductive LD. Furthermore, *GmFT2a* expression in Heihe27 was significantly greater under high temperatures rather than low temperatures when grown with the same photoperiod conditions, suggesting that high temperatures may promote *GmFT2a* expression in the photoperiod-insensitive cultivar. Hence, the following hypothesis was proposed: 1) for a photoperiod-insensitive cultivar, temperature plays a dominant role in *GmFT2a* expression and SD conditions downregulate *GmFT2a* expression; 2) for a photoperiod-sensitive cultivar, photoperiod plays a dominant role in *GmFT2a* expression and high temperature downregulates *GmFT2a* expression. Halliday et al. [29] reported that the *Arabidopsis* phytochrome *phyB* mutant has a temperature-sensitive precocious-flowering phenotype. Zhang et al. [53] suggested that the blue light receptor GmCRY1a plays an important role in photoperiodic flowering in soybean. Therefore, temperature and photoperiod interactively regulate *GmFT2a* expression, yet the underlying mechanism needs further investigation. Future studies utilizing more cultivars with diverse photoperiod sensitivities should prove useful in the unraveling of the relationship between temperature and photoperiod and their influence on flowering.

## Materials and Methods

### Plant materials

This study utilized two soybean cultivars: Zigongdongdou, a photoperiod-sensitive late-flowering cultivar and Heihe27, a photoperiod-insensitive early-flowering cultivar. Soybean seeds were planted in soil in 10-liter pots and grown under LD (16 h light/8 h dark) conditions using 25°C as a default temperature. After seedling emergence, similar pots were produced by removing some seedlings until each pot contained 10 uniform plants. These uniform plants were grown until the cotyledons opened or until the unifoliates fully expanded. The plants were then treated with different photoperiods (LD and SD, i.e., 12 h light/12 h dark) and temperatures (30°C, 25°C and 20°C). Additional details of plant growth and treatments were as reported by Wu et al. [25].

*Arabidopsis thaliana* ecotype Col and the mutant *ft10* (a gift from Dr. Detlef Weigel, Max Planck Institute) were also used. *Arabidopsis* seeds were grown under LD or SD and grown at 22°C.

### Grafting

Zigongdongdou and Heihe27 plants used as scions or stocks were raised under LD conditions until the 11th day after the cotyledons opened (at this time, the first trifoliate had fully expanded). A 5-cm scion was excised, beveled and defoliated with the exception of the newly expanded trifoliate. A stock was beveled at the first trifoliate node with the first trifoliate leaf reserved. The scion was fastened to the stock using Parafilm. To prevent dehydration during the first 5 days after grafting, the scion was covered with a transparent plastic bag and sealed with Parafilm.

### RNA isolation and mRNA purification

Total RNA was isolated using TriZol reagent (Invitrogen). mRNA was purified using the Oligotex™ mRNA Purification Kit (QIAGEN).

### Cloning of *GmFT*

A suppression subtractive hybridization (SSH) library of the Zigongdongdou cultivar was constructed using the PCR Select™-cDNA Subtraction Kit (Clontech). SD-treated leaves were used as the tester, and LD-treated leaves were included as a driver. An expressed sequence tag (EST), homologous to *FT*, was identified using the PCR-Select™ Differential Screening Kit (Clontech). The full-length *GmFT2a* was cloned into the pGEM-T easy vector (Promega) using the FirstChoice™ RLM-RACE Kit (Ambion).

### Real-time quantitative PCR

Total RNA was digested with RQ1 RNase-Free DNase (Promega), and reverse-transcribed with Oligo dT (Sunbiotech, Beijing) using M-MLV Reverse Transcriptase (Promega). Target genes were detected with specific primers (listed in Supporting Information S1) according to the SYBR Premix Ex Taq™ manual (Takara). Primers for *AtAP1* and *AtSOC1* were those used by Chen et al. [54] and Liu et al. [55], respectively. *GmACTIN* or *AtTUB* was included as a reference for soybean or *Arabidopsis* genes, respectively [56].

### *In situ* hybridization

To synthesize the antisense and sense *GmFT2a* RNA probes, a sense primer (5′-CGGGATCCCATTCAGAGGGAATCTGG-3′, with a *BamHI* restriction site) and an antisense primer (5′-GGAATTCTTCCAATTTACGTATATC-3′ with an *EcoRI* restriction site) were used to amplify the 3′-end *GmFT2a* gene-specific region. The product was subcloned into the pSPT18 vector and transcribed *in vitro* using the Digoxigenin (DIG) RNA Labeling kit (Roche Molecular Biochemicals). For each slide, 100 µL of probe was used to achieve a final concentration of 0.4 ng/µL. Tissue sections (10 µm thick) were collected as shown in Yu et al. [57]. *In situ* hybridization and immunological detection were performed as described by Xu et al. [58].

### Transgenic analysis in *Arabidopsis*

Sense (5′-CGTCTAGAATGCCTAGTGGAAGTAG-3′, with a *XbaI* restriction site) and antisense (5′-ATGAGCTCTTAGTATAACCTCCTTC-3′, with a *SacI* restriction site) primers were used to amplify *GmFT*. The product was subcloned into the binary vector p3301m downstream of the 35 S cauliflower mosaic virus promoter. The resulting plasmid (p3301m-GmFT) was used to transform *Arabidopsis* plants by the floral dip method [59]. Transgenic lines were selected for on MS media supplemented with 10 mg/L glufosinate ammonium (Sigma), then grown to obtain T3 transgenic homozygous seeds for further investigation.

### Transgenic analysis in soybean

Sense (5′-GGTCTAGAAAAATAATTCATAACAAAG-3′, with a *XbaI* restriction site) and antisense (5′-CCGAGCTCTCCAATTTACGTATATCAG-3′, with a *SacI* restriction site) primers were used to amplify *GmFT*. The product was subcloned into the vector pGFPGUSplus, replacing *GFP* [60]. From the resultant vector, a HindIII-EcoRI fragment containing the CaMV35S::GmFT::NOS cassette was isolated and inserted into the binary vector pTF101.1 [61]. This vector was designated pTF101.1-GmFT. pTF101.1-GmFT2a was used to transform Zigongdongdou plants, following the cotyledon-node method described by Flores et al. [62]. After selection on 8 and 4 mg/L glufosinate at the second shoot initiation and shoot elongation stages, respectively, primary transformants were planted and grown in the greenhouse. Identified by daubing leaves with 160 mg/L glufosinate (shown in Supporting Information S1), herbicide-resistant soybean lines and their progenies were subject to molecular and phenotypic analysis.

## Supporting Information

Supporting Information S1 Included are seven sections, that is, *GmFT2a* cDNA sequence (Genbank accession number EU287455), GmFT2a putative protein sequence, The alignment between *GmFT2a* and *Glyma16g26660*, Sequence analysis of *GmFT2a*, Sense probe controls for *in situ* hybridization, Transgenic soybean identified by daubing leaves with glufosinate, and Primers used in the research.
(PDF)
