# Supplementary material for: GmFT2a, a Soybean Homolog of FLOWERING LOCUS T, Is Involved in Flowering Transition and Maintenance
Source: PLoS One. 2011 Dec 14;6(12):e29238. doi: 10.1371/journal.pone.0029238 (PMC3237611; doi:10.1371/journal.pone.0029238)
Supplement: Supporting Information S1 — Included are seven sections, that is, GmFT2a cDNA sequence (Genbank accession number EU287455), GmFT2a putative protein sequence, The alignment between GmFT2a and Glyma16g26660, Sequence analysis of GmFT2a, Sense probe controls for in situ hybridization, Transgenic soybean identified by daubing leaves with glufosinate, and Primers used in the research. (PDF) [file pone.0029238.s001.pdf]

GmFT2a cDNA sequence (Genbank accession number EU287455)

1	AAAATAATTC	ATAACAAAGC	AAACGAGTAT	ATAAGAAAGC	ATAAGCCAAA	TTTTGAGTAA
61	ACTAGTGTGC	ACACTATCCC	**ATG**CCTAGTG	GAAGTAGGGA	TCCTCTCGTT	GTTGGGGGAG
121	TAATTGGGGA	TGTATTGGAT	CCTTTTGAAT	ATTCTATTCC	TATGAGGGTT	ACCTACAATA
181	ACAGAGATGT	CAGCAATGGA	TGTGAATTCA	AACCCTCACA	AGTTGTCAAC	CAACCAAGGG
241	TAAATATCGG	TGGTGATGAC	CTCAGGAACT	TCTATACTTT	GATTGCGGTT	GATCCCGATG
301	CACCTAGCCC	AAGTGACCCC	AATTTGAGAG	AATACCTCCA	TTGGTTGGTG	ACTGATATCC
361	CAGCAACAAC	AGGGGCTAGT	TTCGGCCATG	AGGTTGTAAC	ATATGAAAGT	CCAAGACCAA
421	TGATGGGGAT	TCATCGTTTG	GTGTTTGTGT	TATTTCGTCA	ACTGGGTAGG	GAGACCGTGT
481	ATGCACCAGG	ATGGCGCCAG	AATTTCAACA	CTAAAGAATT	TGCTGAACTT	TACAACCTTG
541	GATTGCCAGT	TGCTGCTGTC	TATTTCAACA	TTCAGAGGGA	ATCTGGTTCT	GGTGGAAGGA
601	GGTTATAC**TA A**AAAAAAGTA	CTTTATATTA	TTGAAAAAAA	TAAAGTAGTA	TAAGCTTCGT	
661	TGAGGGTTTC	AGAAATATTA	ATTGGCAATC	TCCCACACTC	TTTAGTAGTA	AATGAGTGTT
721	TTTCAACTTA	ATTAAACTGA	GCATACAGTG	AAATAAATTG	CTAGCTCAGT	TGGTAGCAGC
781	AAGTACTCTG	CATATACACA	TAAATGAAAC	TGAAGCATCT	AGGTTCATTT	TTCTTATTTG
841	TATTATCAGT	TGAAGAATGT	TAAAGATATC	TGATATACGT	AAATTGGA	

The red text indicates the EST sequence isolated by SSH. The underlined ATG and TAA indicate the start and stop codons, respectively. Between these two codons is the ORF (open reading frame) translated into the protein sequence that follows.

GmFT2a putative protein sequence

```
1       mpsgsrdplv  vggvigdvld  pfeysipmrv  tynnrdvsng  cefkpsqvvn  qprvniggdd
61      lrnfytliav  dpdapspsdp  nlreylhwlv  tdipattgas  fghevvtyes  prpmmgihrl
121     vfvlfrqlgr  etvyapgwrq  nfntkefael  ynlglpvaav  yfniqresgs  ggrrly
```

Shown is the putative protein as translated from the ORF described above.

The alignment between *GmFT2a* and *Glyma16g26660*

```
                1                                                          60
26660     (1)   GTAATATTTTTTAAAAATTGACCCATATATGTAAAATACCCGTCAAGATCTCTTTATTAT
GmFT2a    (1)   ------------------------------------------------------------
                61                                                        120
26660    (61)   TTTGAAAGCGAAAGCATATCACTTCAAACACAATGGAATCGAGGCTATTGACTAAGTATA
GmFT2a    (1)   ------------------------------------------------------------
                121                                                       180
26660   (121)   AATAGAGAAGACTTCATATCGGGGTTCATAATTCATAACAAAGCAAACGAGTATATAAGA
GmFT2a    (1)   ------------------------AAAATAATTCATAACAAAGCAAACGAGTATATAAGA
                181                                                       240
26660   (181)   AAGCATAAGCCAAATTTTGAGTAAACTAGTGTGCACACTATCCCATGCCTAGTGGAAGTA
GmFT2a   (37)   AAGCATAAGCCAAATTTTGAGTAAACTAGTGTGCACACTATCCCATGCCTAGTGGAAGTA
                241                                                       300
26660   (241)   GGGATCCTCTCGTTGTTGGGGGAGTAATTGGGGATGTATTGGATCCTTTTGAATATTCTA
GmFT2a   (97)   GGGATCCTCTCGTTGTTGGGGGAGTAATTGGGGATGTATTGGATCCTTTTGAATATTCTA
                301                                                       360
26660   (301)   TTCCTATGAGGGTTACCTACAATAACAGAGATGTCAGCAATGGATGTGAATTCAAACCCT
GmFT2a  (157)   TTCCTATGAGGGTTACCTACAATAACAGAGATGTCAGCAATGGATGTGAATTCAAACCCT
                361                                                       420
26660   (361)   CACAAGTTGTCAACCAACCAAGGGTAAATATCGGTGGTGATGACCTCAGGAACTTCTATA
GmFT2a  (217)   CACAAGTTGTCAACCAACCAAGGGTAAATATCGGTGGTGATGACCTCAGGAACTTCTATA
                421                                                       480
26660   (421)   CTTTGGTAACTCATTAATTTTGTCCAAGTACTCTTTTTGTTGTTCATATTTATAGTGATT
GmFT2a  (277)   CTTTG-------------------------------------------------------
                481                                                       540
26660   (481)   TTAAATTGTAGTAGTAATTTTTTATACCAAGCTAGAATAATTTTTGTGAGTTTTTCTATT
GmFT2a  (282)   ------------------------------------------------------------
                541                                                       600
26660   (541)   GAAAATATAAAACTTTTTTTAATGGAGAAACTCTCAAAAACTCTTTTAACTTTGTATTCA
GmFT2a  (282)   ------------------------------------------------------------
                601                                                       660
26660   (601)   AACAATCTCATAATTATTAGTAAAAAATTTAAATATGATTTTAGTTTCATTAAAATGAGC
GmFT2a  (282)   ------------------------------------------------------------
                661                                                       720
26660   (661)   AAATTTTATTTTTAATATTTGTAAAAAGAAATTGATTTTCTTTTTCTTTTTCTAAGAATA
GmFT2a  (282)   ------------------------------------------------------------
                721                                                       780
26660   (721)   TGACGTTATTGTTTTTGACCATCCATACTACTATTATATGATATAGCATCCATTAATTGT
GmFT2a  (282)   ------------------------------------------------------------
```

```
                781                                                         840
  26660  (781)  TTGTGTTTAAGGGAAGGTCAAGTACCCTCTCAAGTGGACATGTACATACATGTGAGGACA
GmFT2a   (282)  ------------------------------------------------------------
                841                                                         900
  26660  (841)  CCTTTATAATATTTTCTACATCCCAAAATAAATAGTATTATTTATCTATCTCTTTTTAAT
GmFT2a   (282)  ------------------------------------------------------------
                901                                                         960
  26660  (901)  GTATTATTTATGTATCTTAAAGTAAGTTAGGGATTGAAAATATTGATATCTTATTTTAAA
GmFT2a   (282)  ------------------------------------------------------------
                961                                                        1020
  26660  (961)  GGAATGAAAATCAATCATTTTAGAAAAATTAAAACTAAAAATTTGCTCATTGCAGAAAGT
GmFT2a   (282)  ------------------------------------------------------------
                1021                                                       1080
  26660 (1021)  AAAGTAAAGAAAACCCCACCACTCATTTTATAGGACTTAAAGTCATACTTAAACCTTACT
GmFT2a   (282)  ------------------------------------------------------------
                1081                                                       1140
  26660 (1081)  AAAATTTCAAGAATTGAAATACGGATACAAGCAGTTAATGCAATATATTCTTAGTGCAAT
GmFT2a   (282)  ------------------------------------------------------------
                1141                                                       1200
  26660 (1141)  CGAGGATCATTAGATGTTACAATATAGTAGTAGTTTCTAGCTTAATCCAACTTGATTTCA
GmFT2a   (282)  ------------------------------------------------------------
                1201                                                       1260
  26660 (1201)  TTTCATTTTATTAACAGATTGCGGTTGATCCCGATGCACCTAGCCCAAGTGACCCCAATT
GmFT2a   (282)  -----------------ATTGCGGTTGATCCCGATGCACCTAGCCCAAGTGACCCCAATT
                1261                                                       1320
  26660 (1261)  TGAGAGAATACCTCCATTGGTGAGCCACAAATTTTTGTTTAAACACGCCATGAATGCAAA
GmFT2a   (325)  TGAGAGAATACCTCCATTG-----------------------------------------
                1321                                                       1380
  26660 (1321)  CATATATATGAACTGAACTATACATTTATGAGTTTTTCTAGCTTAATGTTGTAATATTAT
GmFT2a   (344)  ------------------------------------------------------------
                1381                                                       1440
  26660 (1381)  ACCCCTATCTTCTCTCAATGTGTCAGGTTGGTGACTGATATCCCAGCAACAACAGGGGCT
GmFT2a   (344)  --------------------------GTTGGTGACTGATATCCCAGCAACAACAGGGGCT
                1441                                                       1500
  26660 (1441)  AGTTTCGGTACGTATATGATGTTTTTCCATTTTTAAAACCTATAATTTTCTGTGCTTAAA
GmFT2a   (378)  AGTTTCG-----------------------------------------------------
                1501                                                       1560
  26660 (1501)  GTTAAACCTATTCAAACCATTATTTTTAATTTAAAATTAGTGAGTTTTATTTATGTGCAT
GmFT2a   (385)  ------------------------------------------------------------
                1561                                                       1620
  26660 (1561)  ATACTGATAAAAAAAATTAGTTCACATATTGTATTTTTATGTATGAAGTCTATTACTGGC
GmFT2a   (385)  ------------------------------------------------------------
```

```
                      1621                                                        1680
26660       (1621)    TTGGTCTGAACAAGTAAATAATCATATATCAAAACAAGAAAAAATATTTTAAATTAAAGT
GmFT2a      (385)     ------------------------------------------------------------
                      1681                                                        1740
26660       (1681)    ATAATTGAACCAAGTAAAATGCATGCTATTAGTTACCAAACTAATAAGCACAAAAAATAA
GmFT2a      (385)     ------------------------------------------------------------
                      1741                                                        1800
26660       (1741)    AATAAAAACTGATAGAAACATGATTGATTACTTATTCAAATTCACCCTCGCTTGTATTCT
GmFT2a      (385)     ------------------------------------------------------------
                      1801                                                        1860
26660       (1801)    GTGTCAAACGCACAATGTTTGCTTGCTAGCTGTTTAATTTCTTTAACAAATGGACACATA
GmFT2a      (385)     ------------------------------------------------------------
                      1861                                                        1920
26660       (1861)    TGCTAAGTGTATTAGTGATTTATGATTTCTAATTATTTAAATTGATAGGAACTCAGCTTT
GmFT2a      (385)     ------------------------------------------------------------
                      1921                                                        1980
26660       (1921)    TCAAAAACATTATTATTAATTATCTTAACTGACGAAGGTATTAGAAATTTTATTGAACAC
GmFT2a      (385)     ------------------------------------------------------------
                      1981                                                        2040
26660       (1981)    TGATTCCTAAACTCGTAAGTTTTGGAATCAAGTGTGTTATGTGATAATAAGAAGATCCCC
GmFT2a      (385)     ------------------------------------------------------------
                      2041                                                        2100
26660       (2041)    ATTAATAATTATTTTACTGAGTATATATAGTGGAAGGGATTTAAATTATGGATATTCTAC
GmFT2a      (385)     ------------------------------------------------------------
                      2101                                                        2160
26660       (2101)    AAGGTTCCATCAACTTTATCTTAAAAGTTTATCTTTTGTCCTTTGGAGCAGTACATCTCC
GmFT2a      (385)     ------------------------------------------------------------
                      2161                                                        2220
26660       (2161)    TTTCCAACAAATGAATGATGTTCGAGTTTATCAAAATATAAATGTGGTCCAAAATATTTT
GmFT2a      (385)     ------------------------------------------------------------
                      2221                                                        2280
26660       (2221)    AAAATAATTTTTATTTTTTTGATATAATAAATATTCTTTTTTATTGAGTACCTTTTTTAA
GmFT2a      (385)     ------------------------------------------------------------
                      2281                                                        2340
26660       (2281)    TTTATATCTATAATTGTTAGAATTATTATTTTTATTTAATTATATTAGAGAAAAATGATT
GmFT2a      (385)     ------------------------------------------------------------
                      2341                                                        2400
26660       (2341)    TAGTTAATTAAGAGTTAAATCTCCAGTAATACACCCCCCCCCCACCCCCCCCAAAAAAAAA
GmFT2a      (385)     ------------------------------------------------------------
                      2401                                                        2460
26660       (2401)    AGTAGCTTAATTTTAATTCATTGGCTAACACAATGTGAAATACTAATGTTATGTTGAAAC
GmFT2a      (385)     ------------------------------------------------------------
```

```
                2461                                                        2520
 26660  (2461)  GGTGCATGCATGATGTATCAAAGCATGCGTTATGCATGGATGTAGGAATGCTAAATGTGG
GmFT2a   (385)  ------------------------------------------------------------
                2521                                                        2580
 26660  (2521)  ACTGTGTTTTCCATTGACTAGTAATTTGAGACTAATCCTATGATTATGGTCAGGCTTAGT
GmFT2a   (385)  ------------------------------------------------------------
                2581                                                        2640
 26660  (2581)  GACTAATTAAAAAGACTCTCAACAAGTACCTTCTTCAAGGTTCGAATTTTGTTTTATTTT
GmFT2a   (385)  ------------------------------------------------------------
                2641                                                        2700
 26660  (2641)  TAATTTTTATTGCCAGAGAATCCAGCTTACAATCACGCCAACCTTTAAAATGAAGAACAG
GmFT2a   (385)  ------------------------------------------------------------
                2701                                                        2760
 26660  (2701)  ATAGAGATAAGTATATGATACTAGGGCATCATATTGTAAAGTACGATCAGCAAACCTGAT
GmFT2a   (385)  ------------------------------------------------------------
                2761                                                        2820
 26660  (2761)  GATGACCAACATACAATCCCAATTAAAATCGGAGATTATTATGATAGATATTTAAGTTGT
GmFT2a   (385)  ------------------------------------------------------------
                2821                                                        2880
 26660  (2821)  TTCTTTGTTTTTTTTTTTATAAAAAAAATCTTTATACAAAACCAAATATGCATATATATA
GmFT2a   (385)  ------------------------------------------------------------
                2881                                                        2940
 26660  (2881)  TATATATATATATATATATATATATATATATATATATATATATATATATATATATATACA
GmFT2a   (385)  ------------------------------------------------------------
                2941                                                        3000
 26660  (2941)  GTTAGTTATACATGTGTATAAATACACACAAACATACATTTATTTTTATATATACATACA
GmFT2a   (385)  ------------------------------------------------------------
                3001                                                        3060
 26660  (3001)  ATATATAGCAGATACTCAATATTGAAAATTAATTTTTGTTTTGATGTTTCAAAAACGTTT
GmFT2a   (385)  ------------------------------------------------------------
                3061                                                        3120
 26660  (3061)  TTATTGGTTTTAATCTTCATATACAAATATTATTTTTTTAAATGCTTCTGTCATTACTTT
GmFT2a   (385)  ------------------------------------------------------------
                3121                                                        3180
 26660  (3121)  GTGATAATATATGACATGCATCTATATATGTTACATCAACAACCATCCTTAAATAACATG
GmFT2a   (385)  ------------------------------------------------------------
                3181                                                        3240
 26660  (3181)  TAATGTAACATATATAGTCATCGATGCATATTACACATTATCATGAGTGAAGATACAGGT
GmFT2a   (385)  ------------------------------------------------------------
                3241                                                        3300
 26660  (3241)  CATTTAAAAAAAAATCAAATTTATGAGGATTAAAACTGACAAAACAAAAATTGATAGACA
GmFT2a   (385)  ------------------------------------------------------------
```

```
                3301                                                        3360
26660   (3301)  TAGGTTACATTAATTAAGCCATATATATTTACAAGAATTAAGAATATATATGTTTTACCT
GmFT2a   (385)  ------------------------------------------------------------
                3361                                                        3420
26660   (3361)  GATTATTAAGATTCAAAATATGCAACCAAATTAGCCAAATTATGAACCATATAAGCATTG
GmFT2a   (385)  ------------------------------------------------------------
                3421                                                        3480
26660   (3421)  AAGTCTTGTTTAATTTCTCTCTTCCATCAGAACTCAAGTAAGAATATATGTAGCCATCCG
GmFT2a   (385)  ------------------------------------------------------------
                3481                                                        3540
26660   (3481)  AATTCTTAAAGTGCATGCATGATCATGTGCAAAAAGATGAGAAGATTCATTCAGTTCAAG
GmFT2a   (385)  ------------------------------------------------------------
                3541                                                        3600
26660   (3541)  AAAAAAGACACAAAGCAACTACTGTGACTAAAGGAGAATATTCCTATTGAATAAACTACC
GmFT2a   (385)  ------------------------------------------------------------
                3601                                                        3660
26660   (3601)  TTTTGTTGGCAAGATAGCTCTAAAACCTTCATAGGATTTACAGAGTTATAGAACATCATA
GmFT2a   (385)  ------------------------------------------------------------
                3661                                                        3720
26660   (3661)  GAAAAAGCTTCATAACTTGTTGAAGCAAATTTAAATAAGGGGAGTTAAAAGAATAAGAAT
GmFT2a   (385)  ------------------------------------------------------------
                3721                                                        3780
26660   (3721)  TAGATTTGTTTAAACTCCATTTCCAAACATCTTATTGACCAAAACTCAGCATCATATATA
GmFT2a   (385)  ------------------------------------------------------------
                3781                                                        3840
26660   (3781)  ATTCAATAAATTCATCACCTTGACAAAATGTGTTCTTTCTAAGTGAAAAAGAACCCTCCT
GmFT2a   (385)  ------------------------------------------------------------
                3841                                                        3900
26660   (3841)  CCATTTAAACTTTCACTTCAACCATACCCCATCAACCTACTCACCAAGGTAAGCAATCTT
GmFT2a   (385)  ------------------------------------------------------------
                3901                                                        3960
26660   (3901)  AACATCCTTCTCATCTGAAATAAGAAAAATTCCTCTGATTTTTTTAAAGAATTTTGAGAT
GmFT2a   (385)  ------------------------------------------------------------
                3961                                                        4020
26660   (3961)  CCATCATCTCTAGTGAACCCCCAATCTTTCCATAAAAAAAGAAAAAAAAGAAAAATATTC
GmFT2a   (385)  ------------------------------------------------------------
                4021                                                        4080
26660   (4021)  TGCTTCCATCATCTATCTGCTTTTTAAAACCAGAGCTTACCAGTATGTATGAGTGTTTCT
GmFT2a   (385)  ------------------------------------------------------------
                4081                                                        4140
26660   (4081)  GGCTTTCTGCCCTTTGCAGTACGAAATGCTAAGATATTTCCACTGAATTGAAATTACGTG
GmFT2a   (385)  ------------------------------------------------------------
```

```
                4141                                                        4200
26660   (4141)  TTCCCATTTCCTATTTAATTTCCTTAGTTTCTTATCTCTTCAGTTAGAGGATGACACGTA
GmFT2a   (385)  ------------------------------------------------------------
                4201                                                        4260
26660   (4201)  CTTAATTAAAATCAACTCTATCTTCTGATTGCTATTATATTTTTGAAGTATTATTTTTTA
GmFT2a   (385)  ------------------------------------------------------------
                4261                                                        4320
26660   (4261)  ATTTGGAAGAGAGGGAAAAGGACACAAGATTAGGTGATTTGGCTCCTTTCTAAAACGTTA
GmFT2a   (385)  ------------------------------------------------------------
                4321                                                        4380
26660   (4321)  GGACAGCAGTAGTAGGACCATACATGTCTGAACATTATTAATACAATACCTTATCTAAGA
GmFT2a   (385)  ------------------------------------------------------------
                4381                                                        4440
26660   (4381)  AGAAATATAGTACTATTTTATGTTTGACAATCAAACTGAAATATATTATCATTGCATGTA
GmFT2a   (385)  ------------------------------------------------------------
                4441                                                        4500
26660   (4441)  TATAAATCATAAGAGTTTAATGAGCGTCAAAGTAGATCAAGTAGAAATCACATATATATA
GmFT2a   (385)  ------------------------------------------------------------
                4501                                                        4560
26660   (4501)  ACTTTTAATATAATTATTATAAAAATTAATAAATTTATTGATCATATAAAATTATTTTAT
GmFT2a   (385)  ------------------------------------------------------------
                4561                                                        4620
26660   (4561)  ATTGTGGGTATTGTAACGATAAATATGCATTATTTGCAAAGGCCATGAGGTTGTAACATA
GmFT2a   (385)  -----------------------------------------GCCATGAGGTTGTAACATA
                4621                                                        4680
26660   (4621)  TGAAAGTCCAAGACCAATGATGGGGATTCATCGTTTGGTGTTTGTGTTATTTCGTCAACT
GmFT2a   (404)  TGAAAGTCCAAGACCAATGATGGGGATTCATCGTTTGGTGTTTGTGTTATTTCGTCAACT
                4681                                                        4740
26660   (4681)  GGGTAGGGAGACCGTGTATGCACCAGGATGGCGCCAGAATTTCAACACTAAAGAATTTGC
GmFT2a   (464)  GGGTAGGGAGACCGTGTATGCACCAGGATGGCGCCAGAATTTCAACACTAAAGAATTTGC
                4741                                                        4800
26660   (4741)  TGAACTTTACAACCTTGGATTGCCAGTTGCTGCTGTCTATTTCAACATTCAGAGGGAATC
GmFT2a   (524)  TGAACTTTACAACCTTGGATTGCCAGTTGCTGCTGTCTATTTCAACATTCAGAGGGAATC
                4801                                                        4860
26660   (4801)  TGGTTCTGGTGGAAGGAGGTTATACTAAGAAAAAGTACTTTATATTATTGAAAAAA-TAA
GmFT2a   (584)  TGGTTCTGGTGGAAGGAGGTTATACTAAAAAAAAGTACTTTATATTATTGAAAAAAATAA
                4861                                                        4920
26660   (4860)  AGTAGTATAAGCTTCGTTGAGGGTTTCAGAAATATTAATTGGCAATCTCCCACACTCTTT
GmFT2a   (644)  AGTAGTATAAGCTTCGTTGAGGGTTTCAGAAATATTAATTGGCAATCTCCCACACTCTTT
                4921                                                        4980
26660   (4920)  AGTAGTAAATGAGTGTTTTTCAACTTAATTAAACTGAGCATACAGTGAAATAAATTGCTA
GmFT2a   (704)  AGTAGTAAATGAGTGTTTTTCAACTTAATTAAACTGAGCATACAGTGAAATAAATTGCTA
```

```
                  4981                                                        5040
 26660   (4980)   GCTCAGTTGGTAGCAGCAAGTACTCTGCATATACACATAAATGAAACTGAAGCATCTAGG
GmFT2a    (764)   GCTCAGTTGGTAGCAGCAAGTACTCTGCATATACACATAAATGAAACTGAAGCATCTAGG
                  5041                                                        5100
 26660   (5040)   TTCATTTTTCTTATTTGTATTATCAGTTGAAGAATGTTAAAGATATCTGATATACGTAAA
GmFT2a    (824)   TTCATTTTTCTTATTTGTATTATCAGTTGAAGAATGTTAAAGATATCTGATATACGTAAA
                  5101                                                        5160
 26660   (5100)   GTGGAAAATATAACTCGAGCATAAGTTAAAGTGATGGAGTATTTATACTTTTGCTTACTA
GmFT2a    (884)   TTGGA-------------------------------------------------------
                  5161                                                        5220
 26660   (5160)   TCCATGATAGTCTATGGGATTGTGCTATTAACAAGTATGATGAAAACTTTCTTTAGCACT
GmFT2a    (889)   ------------------------------------------------------------
                  5221                                                        5280
 26660   (5220)   AATTGTACTTAAGATTAGCTCTTTAATAAATTAAAATCGTTTAGCTAGCTTATATATATA
GmFT2a    (889)   ------------------------------------------------------------
                  5281                 5302
 26660   (5280)   TATATATAAAACACGACTAGGT
GmFT2a    (889)   ----------------------
```

The soybean genomic fragment aligned here ranges from nucleotide 30741500 to 30746800 of the 16th chromosome, which contains *Glyma16g26660* labeled as *26660*. The letters shaded with gray indicate the putative transcript of *Glyma16g26660*. The letters with gray background are the putative exons while the letters with white background and located between them are the putative introns. Both the putative exons and introns constitute the annotated *Glyma16g26660*. The letters with gray or yellow background are the identity nucleotides between *GmFT2a* and *Glyma16g26660* or its flanking genomic sequences. The letters of increased size indicate the start or stop codons of *GmFT2a*.

Sequence analysis of *GmFT2a*

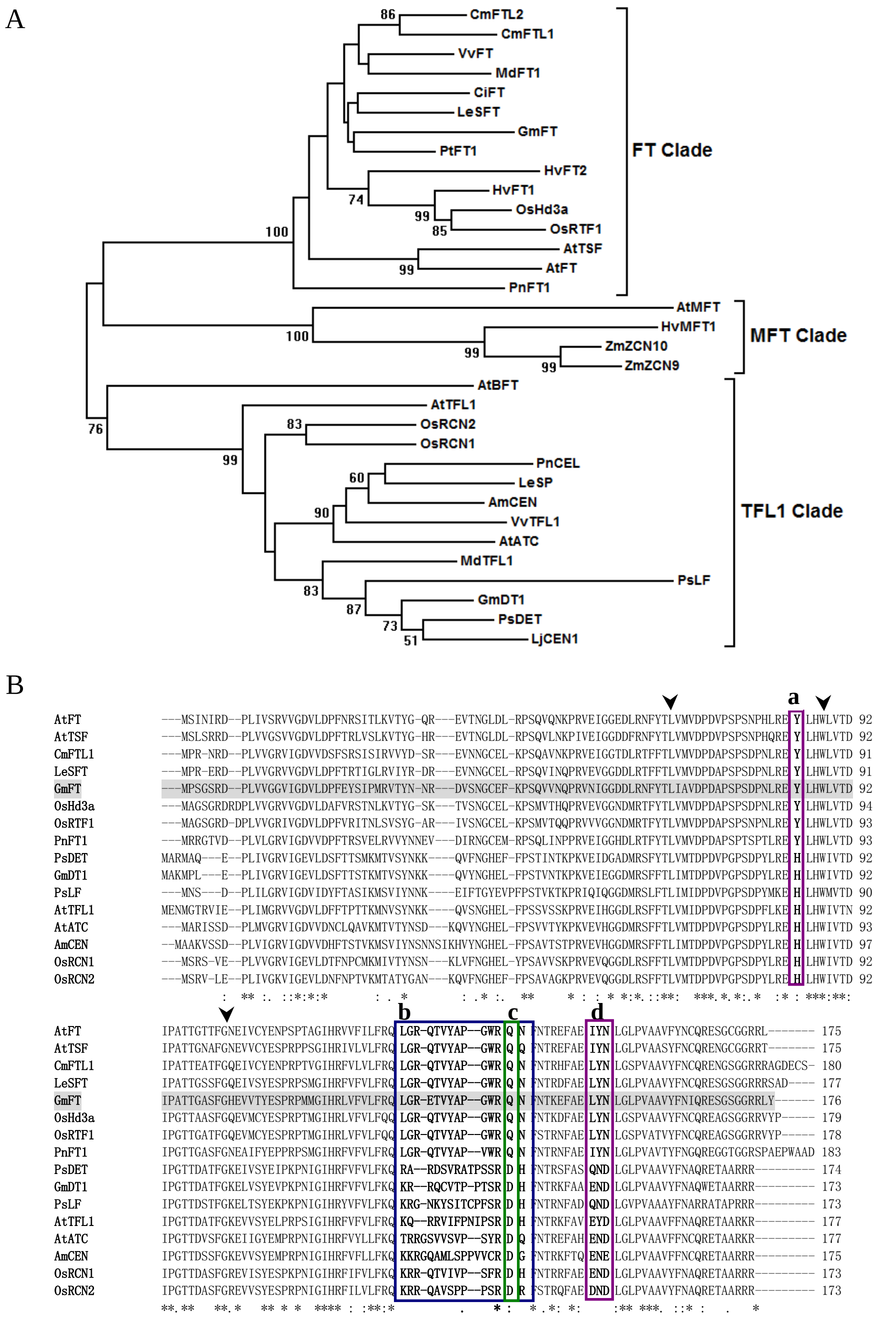

C

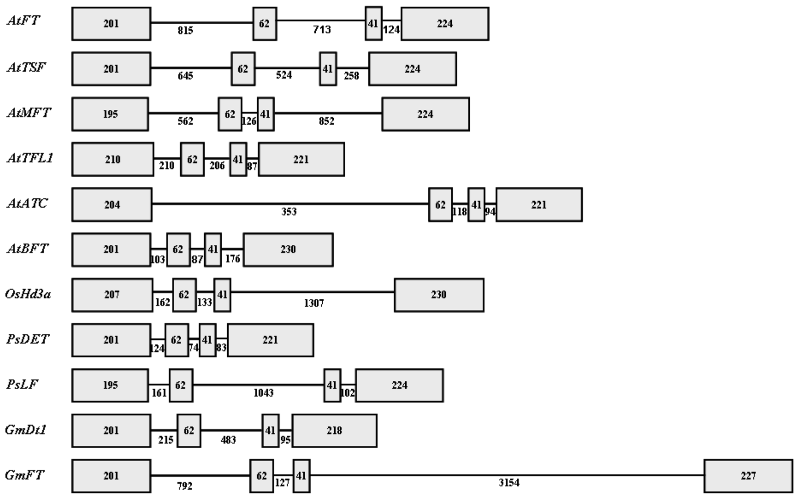

(**A**) An unrooted neighbor-joining phylogenetic tree of GmFT2a and related proteins was constructed by MEGA4.0 with bootstrap values at nodes from 1000 replications. Bootstrap values less than 50% were omitted. Three main clades were also shown. (**B**) Alignment of proteins from FT clade and TFL1 clade by Clustalx version 2.0. The arrow-heads indicate the boundaries between exons. Boxes a, b, c and d show the conserved amino acid residues or segments used to differentiate the activity of FT and TFL1. (**C**) *GmFT2a* has a conserved gene structure. Exons are shown as boxes and introns as lines. The number in the boxes and under the lines indicates the length of the exons and introns, respectively. AmCEN (GenBank accession number AAB36112) is from *Antirrhinum majus*; AtFT (AAB027504), AtTSF (AB027506), AtTFL1 (CAB85504), AtATC (AB024715), AtBFT (Q9FIT4) and AtMFT (AF147721) from *Arabidopsis thaliana*; CiFT (BAA77836) from *Citrus unshiu*; CmFTL1 (ABI94605) and CmFTL2 (ABI94606) from *Cucurbita maxima*; GmDT1 (BAI66119.1) and GmFT2a (GmFT, EU287455) from *Glycine max*; HvFT1 (AAZ38709), HvFT2 (ABB99414) and HvMFT1 (BAH24198) from *Hordeum vulgare*; LeSP (AAC26161.1) and LeSFT (AAO31792) from *Lycopersicon esculentum*; LjCEN1 (AAQ93599) from *Lotus japonicus*; MdFT1 (BAD08340) and MdTFL1 (BAD06418) from *Malus x domestica*; OsHd3a (AB052944), OsRFT1 (BAB78480), OsRCN1 (AF159882), and OsRCN2 (BAH01422) from *Oryza sativa*; PnFT1 (ABW73562)and PnCEL (BAE44112) from *Pharbitis nil*; PsLF (AAQ20811) and PsDET (AAR03725) from *Pisum sativum*; PtFT1 (ABD52003) from *Populus tremula*; VvFT (ABI99465) and VvTFL1 (AAM46142)from *Vitis vinifera*; ZmZCN9 (NP_001106248) and ZmZCN10 (NP_001106249) from *Zea mays*.

Sense probe controls for *in situ* hybridization

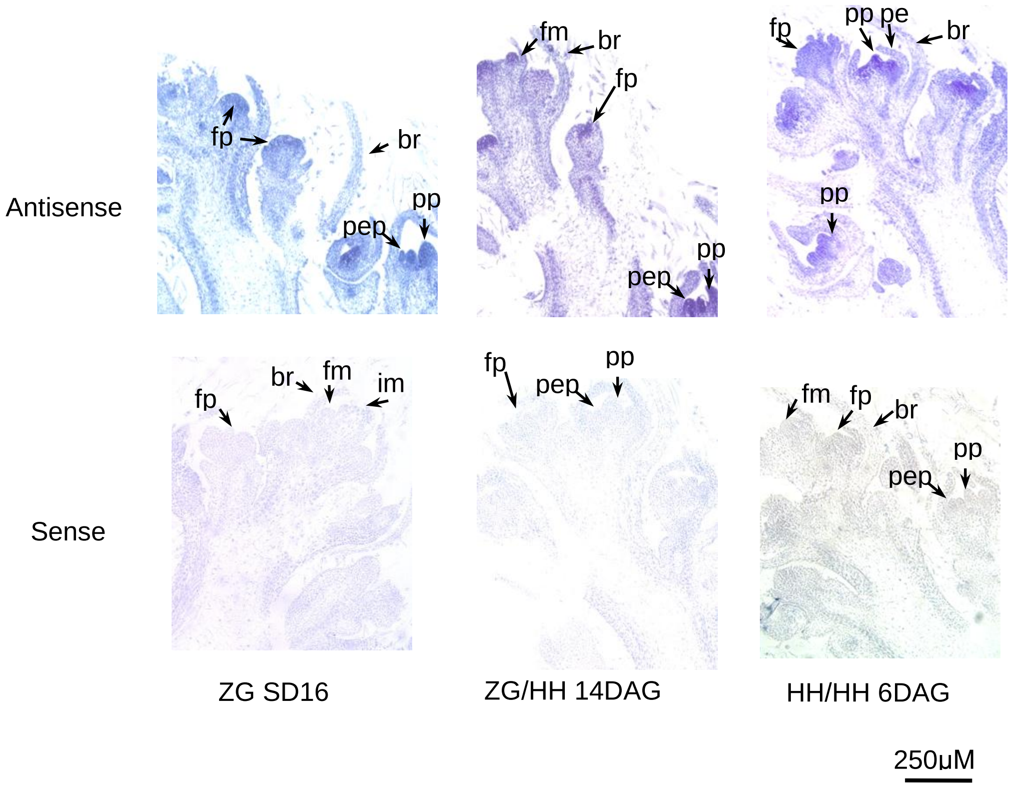

GmFT2a transcripts in the shoot apices were detected by the antisense (the upper row) and sense probes (the lower row). ZG SD16, Zigongdongdou under SD for 16 days; ZG/HH 14DAG, scion Zigongdongdou grafted on stock Heihe27 (ZG/HH) at 14 DAG; and HH/HH 6DAG, self-grafted Heihe27 at 6 DAG. br, bract; fm, floral meristem; fp, floral primordium; im, inflorescence meristem; pe, petal; pep, petal primordium; pp, pistil primordium.

Transgenic soybean identified by daubing leaves with glufosinate

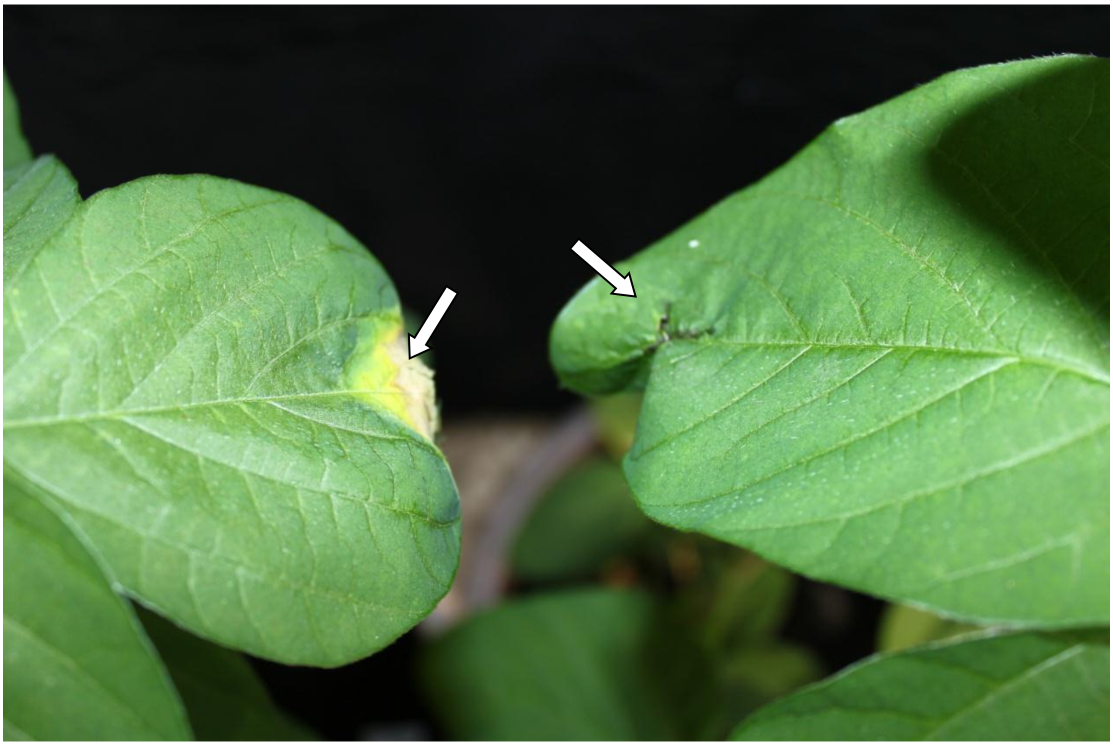

CK **_35S::GmFT2a_**

White arrows show where leaves from wildtype and transgenic plants were daubed with glufosinate. CK, wildtype; *35S::GmFT2a*, transgenic.

Primers used in the research

Name	Sequence
GmFT2a qPCR primers	Sense: 5'TAATTCATAACAAAGCAAACGAGTA 3'<br>Antisense: 5' GCTGACATCTCTGTTATTGTAGGTA 3'
AtFT qPCR primers	Sense: 5' CCTGCTACAACTGGAACAACC 3'<br>Antisense: 5' CAGCCACTCTCCCTCTGACAAT 3'
GmACTIN qPCR primers	Sense: 5' GAGCAAGAACTCGAGACTGCAA 3'<br>Antisense: 5' TTCCAGCAGCTTCCATTTCA 3'
AtAP1 qPCR primers [1]	Sense: 5'-ACAATATGCCTCCCCCTC-3'<br>Antisense: 5'-CTTCTTGATACAGACCACCC-3'
AtSOC1 qPCR primers [2]	Sense: 5'-AGCTGCAGAAAACGAGAAGCTCTCTG-3'<br>Antisense: 5'-GGGCTACTCTCTTCATCACCTCTTCC-3'
AtFLC qPCR primers	Sense: 5' CTTCTCGTCGTCTCCGCCTC 3'<br>Antisense: 5' CAGTCTCAAGATGTTCCTCCAG 3'
AtTUB qPCR primers	sense: 5' AAACTCACTACCCCCAGCTTTG 3'<br>Antisense: 5'CACCAGACATAGTAGCAGAAATCAAGT 3'

1. Chen NZ, Zhang XQ, Wei PC, Chen QJ, Ren F, et al. (2007) AtHAP3b plays a crucial role in the regulation of flowering time in Arabidopsis during osmotic stress. J Biochem Mol Biol 40: 1083-1089.
2. Liu C, Zhou J, Bracha-Drori K, Yalovsky S, Ito T, et al. (2007) Specification of Arabidopsis floral meristem identity by repression of flowering time genes. Development 134: 1901-1910.
